# Is MG53 a potential therapeutic target for cancer?

**DOI:** 10.3389/fendo.2023.1295349

**Published:** 2023-11-15

**Authors:** Yunyu Du, Tieying Li, Muqing Yi

**Affiliations:** ^1^ School of Sports Science, Beijing Sport University, Beijing, China; ^2^ National Institute of Sports Medicine, Beijing, China

**Keywords:** MG53, cancer, glucose metabolism, membrane repair, insulin resistance

## Abstract

Cancer treatment still encounters challenges, such as side effects and drug resistance. The tripartite-motif (TRIM) protein family is widely involved in regulation of the occurrence, development, and drug resistance of tumors. MG53, a member of the TRIM protein family, shows strong potential in cancer therapy, primarily due to its E3 ubiquitin ligase properties. The classic membrane repair function and anti-inflammatory capacity of MG53 may also be beneficial for cancer prevention and treatment. However, MG53 appears to be a key regulatory factor in impaired glucose metabolism and a negative regulatory mechanism in muscle regeneration that may have a negative effect on cancer treatment. Developing MG53 mutants that balance the pros and cons may be the key to solving the problem. This article aims to summarize the role and mechanism of MG53 in the occurrence, progression, and invasion of cancer, focusing on the potential impact of the biological function of MG53 on cancer therapy.

## 1 Introduction

The tripartite-motif (TRIM) family is characterized by a really interesting new gene (RING) finger domain, one or two B-box domains, and a coiled coil domain (1). Tripartite domains are highly conserved among TRIM proteins and hence perform similar functions in cellular processes (2). The vast majority of TRIM proteins contain RING finger domains in their N-terminal regions and seem to participate mostly in ubiquitination (3). B-box domains may exist solely in TRIM proteins and may mediate protein–protein interactions (1, 4). The coiled coil domain has been proven to mediate homo-oligomeric and hetero-oligomeric interactions given that self-association via this domain is believed to play a critical role in catalytic activity of TRIM proteins (5). The variation in the C-terminal domain contributes to the diverse functions of TRIM proteins.

About 80 TRIM protein genes have been identified in humans (6). Many diseases have been shown to be associated with TRIM proteins. These diseases include metabolic and neurodegenerative diseases, viral infections, and cancers (7–10). The role of TRIM proteins in cancer has received more attention. As a result of structural differences, TRIM proteins act as oncogenes and tumor suppressors in different cancers (11). However, the relationship between some members of TRIM proteins and cancer remains unexplored (10).

TRIM72, also known as Mitsugumin 53 (MG53), is secreted by muscle tissues and is a TRIM family protein derived from an immunoproteomics pool (12). The C-terminal of MG53 contains PRY and SPRY domains, which are the most common domains in TRIM proteins (4, 13). These domains can recognize specific partner proteins, thus acting as protein-interacting modules (14). As a typical E3 ubiquitin ligase, MG53 was initially found to participate in damage repair in skeletal muscle cells, and its key feature of membrane repair in a variety of organ injuries was later confirmed (12, 13). MG53 overexpression can inhibit systemic insulin response and subsequently cause metabolic issues (15). However, other researchers take a completely opposing position (16). Evidence suggesting that MG53 may perform an anticancer role in cancers, such as hepatocellular carcinoma, colorectal carcinoma, tongue cancer, and nonsmall cell lung cancer (NSCLC), has recently emerged (17–20). In this review, we summarize the roles and mechanisms of MG53 in a variety of cancers and discuss the possible contribution of the diverse biological functions of MG53 in cancer.

## 2 Beneficial effects of MG53 on cancer therapy

### 2.1 MG53 in colorectal carcinoma

Colorectal cancer is the second most common cause of cancer deaths worldwide and is expected to cause 1.2 million deaths by 2030 (21, 22). Considering that most patients with colorectal cancer progress slowly over many years, colorectal cancer is usually curable if diagnosed at an early stage (23). Screening for colorectal cancer requires the development of sensitive biomarkers in peripheral blood. Many members of the TRIM protein family have been reported to act as oncogenic and tumor-suppressive factors in gastrointestinal cancers via different signaling pathways (24). In addition, TRIM47 may be an effective diagnostic marker for predicting colorectal cancer (25).

The gene and protein levels of MG53 were considerably lower in colon cancer tissues than in healthy colon tissues, and the same results were found in the serum of patients with colon cancer (26). In colon cancer and normal colon tissues, MG53 may be expressed and secreted by stromal cells instead of normal colon or colon cancer cells, and serum MG53 levels are negatively correlated with colon cancer stage and metastasis, suggesting that the low MG53 levels in the serum of patients with colon cancer may be due to local tissue lesions (26). Low levels of MG53 in focal tissues have also been suggested to account for the poor prognosis of stage II colon carcinoma (27). Under colorectal carcinogen induction, MG53 knockout mice present more severe tumor progression than wild-type mice, whereas mice with MG53 overexpression have relatively good colorectal structure and function (19). MG53 has also been shown to inhibit the proliferation of colorectal cancer cells in an *in vitro* study. And this study found that MG53, as an E3 ubiquitin ligase capable of targeting cyclin D1, induces its ubiquitination-dependent degradation to inhibit the proliferation of gastrointestinal cancer cells by arresting the cell cycle at the G1 phase (28). In addition, MG53 acts differently on different anticancer drugs. MG53 and pabocinib inhibit the proliferation of colon cancer cells synergistically, and MG53 could partially ameliorate drug resistance (19). The safety of recombinant human MG53 (rhMG53) has been validated in a mouse model of colorectal cancer (28). Although rhMG53 do not affect the doxorubicin sensitivity of resistant colorectal cancer cells (SW620/AD300), it inhibits the proliferation of colorectal cancer cells. Moreover, in mouse tumor xenograft models of colorectal adenocarcinoma with multidrug resistance, the combination of doxorubicin and rhMG53 appeared to be more effective than doxorubicin or rhMG53 alone (28).

### 2.2 MG53 in hepatocellular carcinoma

Although vaccination and antiviral therapy have reduced the incidence of hepatocellular carcinoma, the incidence and mortality rates of this malignancy continue to increase in many regions of the world (29). In hepatocellular carcinoma, the expression of numerous TRIM proteins tends to be altered and has been shown to be correlated with diagnosis, treatment, and prognosis (30). TRIM proteins appear to be involved in the survival, growth, aerobic glycolysis, immune infiltration, and invasion of hepatocellular carcinoma cells (31–34).

The mRNA expression of MG53 was detected in human hepatocellular carcinoma and normal human hepatocyte cell lines. In patients with hepatocellular carcinoma, the high expression of MG53 may be associated with poor overall survival (35). However, one study has shown that the gene and protein expression levels of MG53 have been suggested to be drastically lower in hepatocellular carcinoma tissue than in matched noncancerous liver tissue (17). MG53 regulates the ubiquitination and degradation of RAC1, a small GTPase with oncogenic function, this effect, in turn, inhibits the malignant progression of hepatocellular carcinoma and improves the resistance of hepatocellular carcinoma to sorafenib treatment by blocking the RAC1/MAPK signaling pathway (17).

### 2.3 MG53 in NSCLC

Although the application of precision medicine in NSCLC treatment has advanced considerably over the past decade, the 5-year survival rate of patients with metastatic NSCLC remains less than 5% due to multiple drug resistance mechanisms (36, 37). Some TRIM proteins may contribute to NSCLC or resistance to targeted drugs (38–44), whereas others have completely opposite functions (45–47).

MG53 is downregulated in metastatic tumors from patients with NSCLC relative to in nonmetastatic tumors, and MG53 knockout promotes the growth and metastasis of lung tumors in mice (48, 49). G3BP2, a protein associated with the formation of multiple tumors, was upregulated in the cytosol of tumor cells from patients with NSCLC relative to in nontumor cells. Circulating levels of MG53 appear to influence the proliferation and migration of NSCLC cells directly via G3BP2. Instead of performing classical ubiquitination-dependent degradation functions, the amino terminus of MG53 physically interacts with G3BP2 and enhances its nuclear translocation, which may be a key mechanism by which MG53 inhibits the G3BP2-mediated formation of lung cancer tumors and stress granules (20, 50). Furthermore, an *in vitro* study showed that rhMG53 inhibited the formation of stress granules and potentiated the cytotoxic effect of cisplatin on human NSCLC cells (20).

### 2.4 MG53 in other cancers

MG53 appears to have an ameliorative effect on multiple types of cancer. However, many TRIM proteins have inconsistent effects on different cancers. A three-dimensional growth system study reported that MG53 dramatically suppressed the proliferation, invasion, and colony formation of tongue cancer cells (18). Knocking down MG53 in tongue cancer cells resulted in a remarkable increase in the phosphorylation of AKT^Ser308^ and AKT^Thr473^. Animal studies showed that in mice, knocking out MG53 also accelerated the progression of tongue cancer (18). O6-methylguanine DNA methyl transferase (MGMT) is an important target in cancer therapy because it blocks the beneficial effects of chemotherapy on tumor cells (51). The RING structural domain of MG53 interacts with the N-terminal region of MGMT and regulates the ubiquitination-dependent degradation of MGMT. Human uveal melanoma cells have higher MGMT levels and lower MG53 levels than normal human pigment epithelium cells. MG53 overexpression in uveal melanoma cells contributes to improved chemoresistance to dacarbazine treatment (52). MG53 is downregulated in the tumor tissue of patients with breast cancer relative to in paired adjacent nontumor tissue and is also downregulated in many breast cancer cell lines relative to in normal human mammary cell lines. *In vivo* and *in vitro*, MG53 inhibits breast cancer progression likely because it can inhibit the activation of the PI3K/Akt/mTOR pathway and reduce lactate levels through protein phosphatase 3 catalytic subunit α (53). One study analyzed ubiquitin-related genes in The Cancer Genome Atlas cohort and found that MG53 was correlated strongly with the grade, stage, and T stage of clear cell renal cell carcinoma. However, the expression of MG53 in patients with clear cell renal cell carcinoma remains to be confirmed (54).

**Figure 1:**
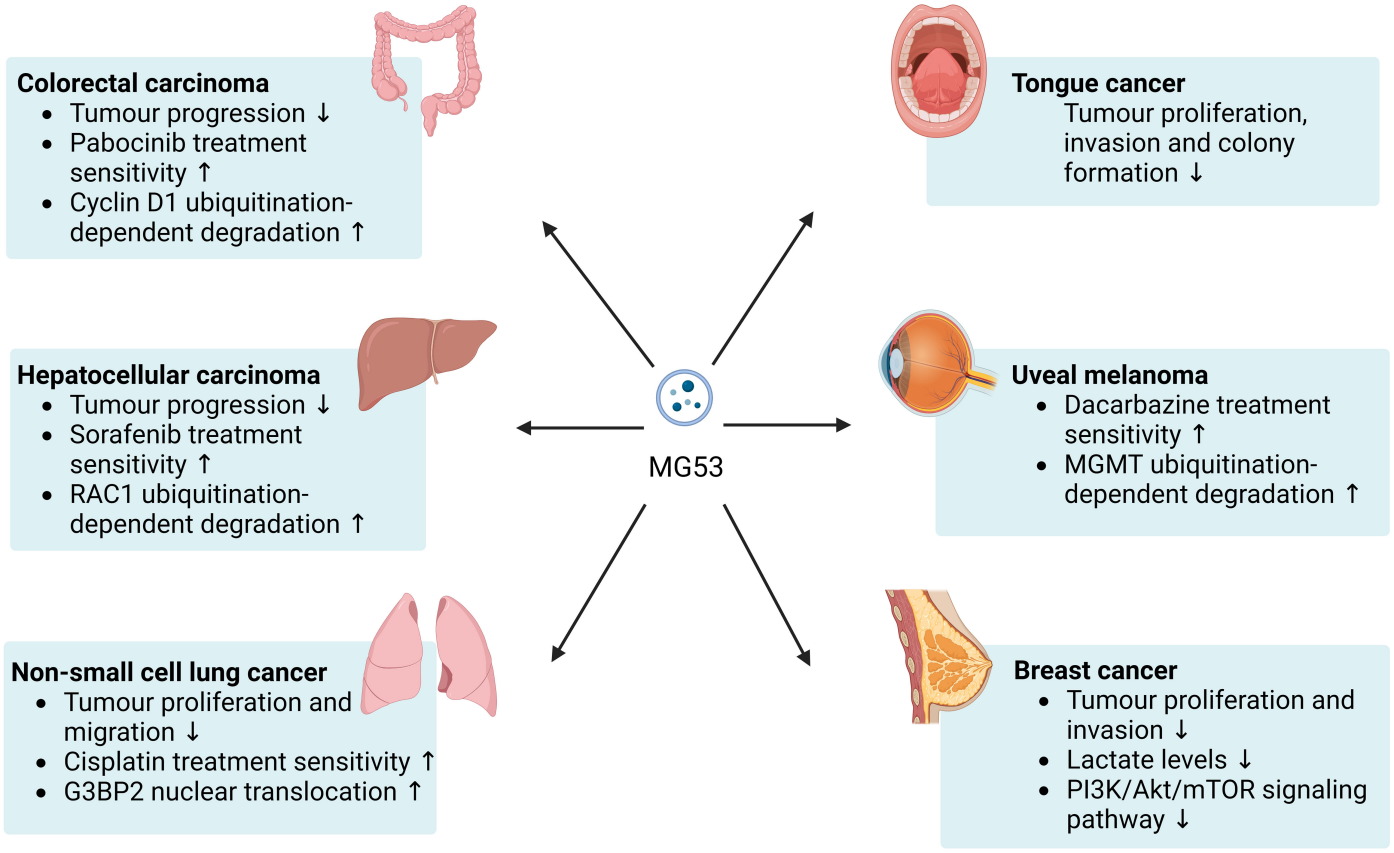
Beneficial effects of MG53 on cancer. MG53 can inhibits the progression of a broad range of cancers and helps improve the therapeutic sensitivity of numerous anticancer drugs. The antitumor capacity of MG53 may be mainly attributed to its E3 ubiquitin ligase properties.

In accordance with the current evidence, MG53 appears to be beneficial for delaying the progression of various cancers and improving resistance to some anticancer drugs in *in vitro* and animal models (**Figure 1**). Available studies suggest that the antitumor effect of MG53 may be mainly derived from its role as an E3 ubiquitin ligase. However, the current evidence for specific cancer types remains insufficient and lacks mechanism research. Further safety verification is required for the application of rhMG53.

## 3 Other biological functions of MG53 may contribute to cancer therapy

### 3.1 Potential role of MG53 as a plasma membrane repair protein in cancer treatment

During cancer progression and treatment, many organs suffer varying degrees of tissue damage from the tumor, cancer complications, and treatment side effects, all of which are related to plasma membrane damage and may accelerate cancer progression (55–57). MG53 was initially well known for its function in the repair of muscle cell membranes. Evidence showing that MG53 can participate in the repair of various cell membranes and promote tissue regeneration has emerged with the deepening of research (13, 58). MG53 secreted by skeletal muscles is transported in the circulatory system in the form of vesicles and participates in muscle cell membrane repair. The failure of MG53-mediated membrane damage repair may cause certain skeletal muscle diseases (12, 59–61). In addition, the pathological processes of myocardial injury and cancer are intertwined, and heart failure induced by anticancer therapy has become a key focus in cardiac oncology research (62, 63). Evidence also suggests that cancer and ischemia–reperfusion injury share common pathways also exists (64).

#### 3.1.1 MG53 in kidney injury

The occurrence of acute and chronic kidney injury is strongly associated with the development of kidney cancer, and early intervention for kidney injury is an effective means of kidney cancer prevention (65). Moreover, the presence of acute kidney injury is fairly prevalent in patients with cancer. The management strategies for acute kidney injury differ in accordance with predisposing factors. For example, immunotherapy-induced acute kidney injury is influenced by tumor type and treatment modality (66, 67). When renal proximal tubular epithelium cells experience acute injury, such as mechanical or chemical damage, MG53 rapidly translocates to the injured site to form a repair patch. By contrast, in injured renal proximal tubular epithelium cells with MG53 knockout, the defect in membrane repair function leads to rapid death of cells. MG53 knockout mice exhibit tubulointerstitial defects and show more severe renal injury than wild-type mice during ischemia–reperfusion. In animals, the preadministration of rhMG53 alleviates cisplatin or iodine contrast agent-induced acute kidney injury (68, 69). In chronic kidney disease, MG53 provides benefits by controlling inflammation and promoting mitochondrial autophagy (70, 71).

#### 3.1.2 MG53 in lung injury

Chronic lung injury, such as chronic obstructive pulmonary disease, is strongly associated with the development of lung cancer, and this mechanistic overlap has attracted increasing attention (72, 73). Chemotherapy, surgery, medication treatment for lung cancer, and even treatment for other types of cancer can lead to lung injury (74–77). Chronic moderate liver injury tends to induce hepatic cell carcinogenesis rather than hepatocellular senescence, which can inhibit carcinogenesis (78). In several models of lung injury, MG53 shows reparative effects on pulmonary epithelial cells. Animals lacking MG53 exhibit increased susceptibility to injury induced by various factors, and rhMG53 can protect lung tissue from lung injury. MG53 may execute its membrane repair function by coregulating the endocytosis of alveolar epithelial cells with caveolin 1 (79–85).

#### 3.1.3 MG53 in liver injury

The liver is susceptible to the effects of drugs, such as conventional chemotherapy drugs, small-molecule-targeting drugs, including multikinase inhibitors, or immune checkpoint inhibitors, all of which can induce varying degrees of liver injury (86–88). With the widespread application of immune checkpoint inhibitors in liver tumor therapy, the relationship between checkpoint inhibitors and liver safety has received increased attention (89). Although hepatocytes do not express MG53 mRNA, circulating MG53 leads to the ubiquitination-dependent degradation of RIPK3, which inhibits the phosphorylation and membrane translocation of MLKL and thus alleviates acetaminophen-induced hepatocyte injury (90, 91). MG53 can also ameliorate oxidative stress and hepatocyte death induced by hepatic ischemia–reperfusion through interaction with dysferlin (92).

Overall, the plasma membrane repair function of MG53 has considerable potential for application in cancer prevention and treatment. Current research focuses on the association between MG53 and the progression of tumor tissues, whereas only a few studies have investigated the contribution of plasma membrane repair by MG53 to cancer treatment. However, the fact that excessive membrane repair contributes to cancer cell invasion is also important to consider when using rhMG53 (93). For example, the annexin family, which participates in membrane repair together with MG53, is overexpressed in invasive cancer cells and promotes the plasma membrane repair of cancer cells. Inhibiting Annexin-mediated repair is beneficial for inducing cancer cell death (94–98).

### 3.2 Potential role of MG53 as an anti-inflammatory factor in cancer treatment

Inflammatory response is an important defense mechanism of the body, but it can also promote the formation of tumor microenvironment and tumor promotion, especially chronic inflammation (99). Anti-inflammatory therapy targeting inflammation-related factors such as nuclear factor-κB (NF-κB) plays an important role in cancer control (100). TRIM proteins are widely involved in regulating inflammatory responses and MG53 appears to have anti-inflammatory effects in multiple tissues (101).

MG53 interacts with the p65 subunit of NF-κB and thereby inhibits the nuclear translocation of NF-κB, which in turn alleviates inflammatory responses in kidney, nervous system and airway (70, 102, 103). After infection of macrophages or mice with virus, MG53 attenuates inflammatory response by decreasing type I interferon levels (104). In mice with Duchenne muscular dystrophy, MG53 appears to enhance mitochondrial autophagy, thereby reducing nucleotide oligomerization domain-like receptor protein 3 (NLRP3) inflammasomes and suppressing chronic inflammation in skeletal muscles (105). Similarly, it was emphasised that MG53 may improve neuroinflammation by decreasing NLRP3 inflammasomes in a study using human umbilical cord mesenchymal stem cells and mice (106).

MG53 can ameliorate inflammation in many disease models, but its role in carcinogenic inflammation, inflammation caused by cancer and inflammation triggered by cancer treatment remains to be investigated.

Furthermore, other biological functions of MG53 may be beneficial for cancer therapy. For example, angiogenesis is an important target for cancer treatment, and MG53 inhibits angiogenesis *in vivo* and *in vitro* by decreasing focal adhesion kinase phosphorylation and blocking the Src/Akt/ERK1/2 signaling pathway (107, 108). Peroxisome proliferator-activated receptor-α (PPARα) agonists have a role in anti-tumor therapy and MG53 attenuates inflammatory responses in cardiomyocytes by upregulating PPARα expression (109, 110). However, there is too little relevant evidence to demonstrate that these functions of MG53 are beneficial for cancer therapy.

## 4 Potential adverse effects of MG53 on cancer treatment

### 4.1 MG53 overexpression may disrupt glucose metabolism signals

Insulin resistance is a key factor in the occurrence and development of cancer, and a substantial proportion of patients with cancer have insulin resistance (111–114). Impaired glucose tolerance is also strongly associated with long-term cancer risk and is an important risk factor for cancer-related death (115–119). During cancer treatment, the blood glucose and insulin levels of patients must be monitored to learn about the insulin resistance induced by therapeutic measures and thus adjust the treatment protocol promptly (120).

The relationship between MG53 and insulin resistance has long been controversial. Some studies have suggested that MG53 induces insulin resistance through multiple pathways, including targeting insulin receptor substrate 1 (IRS-1), insulin receptors (IRs), and AMP-activated protein kinase for ubiquitin-dependent degradation, promoting the expression of peroxisome proliferator-activated receptor-α and its target genes to facilitate myocardial lipid uptake and thereby leading to lipid accumulation and toxicity, and binding to the extracellular structural domains of IRs to inhibit receptors allosterically (15, 121–124). In addition, the direct application of rhMG53 may exacerbate insulin resistance, and the protective effect of MG53 on myocardial cells may be counteracted by its adverse metabolism. Two mutants of rhMG53, rhMG53-C14A and rhMG53-S255A, can eliminate adverse effects on metabolism while retaining the membrane repair function of rhMG53 (121, 125–128).

However, MG53 expression is inconsistent in various models of metabolic disorders, and neither the ablation nor overexpression of MG53 in wild-type and db/db mice has been noted to alter insulin signaling. Additionally, in rats, the repeated intravenous administration of rhMG53 does not seem to affect glucose metabolism (16, 129–133). Indeed, the lack of IRS-1 does not immediately give rise to diabetes because strong compensatory mechanisms exist between different IR subtypes (134, 135).

**Figure 2:**
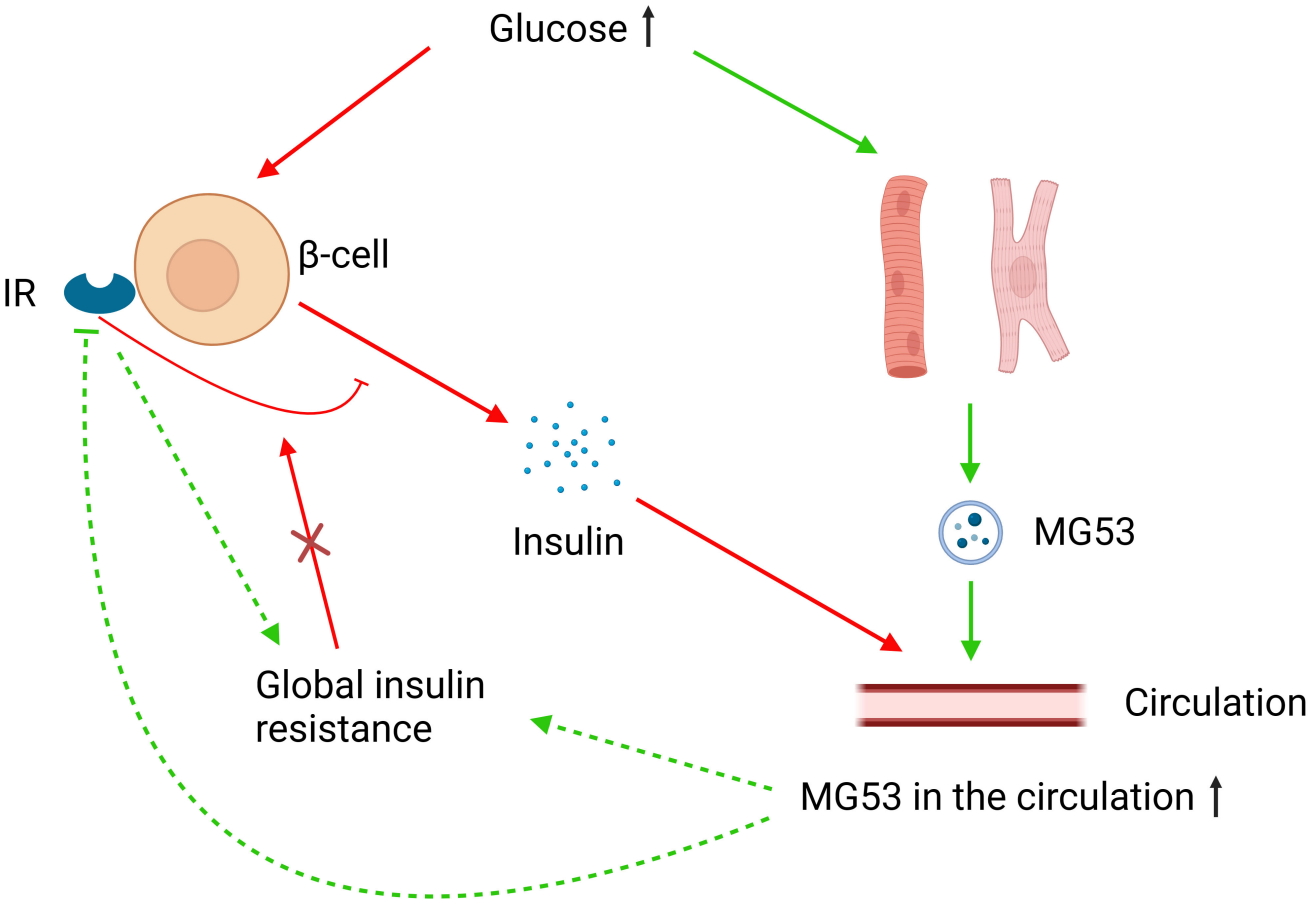
MG53 and glucose metabolism. Elevated glucose levels increase MG53 secretion from muscle tissue, and excess MG53 can lead to global insulin resistance through the inhibition of IRs or other pathways. High glucose also stimulates insulin secretion from pancreatic β-cells, where IRs play an inhibitory role. However, in the case of global insulin resistance, this inhibitory function of IRs fails. MG53 may have different effects on insulin secretion and therefore glucose metabolism in different severities of insulin resistance.

The aforementioned controversy may be attributed to the overlooked role of MG53 in pancreatic β-cells. In the absence of global insulin resistance, the IRs of pancreatic β-cells can inhibit high glucose-induced insulin secretion and their knockout can promote insulin secretion and improve glucose tolerance. However, this regulatory function of the IRs to β-cells does not occur in the presence of global insulin resistance (136). High glucose and insulin levels can promote the secretion of MG53 in striated muscle, and MG53 can induce the ubiquitination-dependent degradation and inactivation of IRs (15, 121). Under the assumption that MG53 can affect the function of pancreatic β-cells through IRs, MG53 overexpression would have a complicated effect on glucose metabolism in healthy and insulin-resistant humans (**Figure 2**). A cohort study involving 283 subjects supports our hypothesis. This study found that although serum MG53 levels appeared to be unrelated to insulin resistance, subjects with impaired glucose metabolism had remarkably higher circulating levels of MG53 than healthy subjects. Furthermore, circulating levels of MG53 were found to be an independent risk factor for the development of type 2 diabetes rather than a simple disease marker, and elevated circulating levels of MG53 represent the diminished function of β-cells (137).

The above evidence suggests that MG53 has important implications for glucose metabolic disorders although the relationship between MG53 and insulin resistance is controversial. However, current research remains insufficient to elucidate its underlying mechanisms. Additional robust evidence is needed to explain the mechanism underlying the involvement of MG53 in glucose metabolism and validate the safety of rhMG53 in patients with metabolic disorders and cancer.

### 4.2 MG53 inhibits myogenesis and promotes myocardial fibrosis

The potential adverse effects of MG53 on cancer cachexia must also be considered when applying rhMG53 in cancer therapy. Cancer cachexia, a common syndrome among patients with cancer, is characterized primarily by the loss of muscle tissue and inadequately relieved by nutritional means (138, 139). Changes in factors related to protein metabolism during cancer progression or treatment led to an imbalance between protein synthesis and degradation, resulting in muscle tissue reduction (140, 141). Reduced muscle mass in cancer cachexia is partly attributed to suppression of the anabolic signaling pathway induced by insulin-like growth factor1 (140). Cardiac atrophy and fibrosis in cancer cachexia are associated with the activated transforming growth factor-β (TGF-β)-mediated SMAD2/3 catabolic signaling pathway (142–145). MG53 inhibits the IGF-induced IRS-1/PI (3)K/Akt pathway, which is the best-characterized mechanism in cardiac and skeletal muscle myogenesis, through the ubiquitin-dependent degradation of IRS-1 (125, 139, 146, 147). Caveolin-1 plays an antifibrotic role in multiple organs and reduces cardiac fibrosis by repressing the TGF-β/Smad2 pathway (148, 149). MG53 can inhibit the expression of caveolin-1, thereby promoting TGF-β1/SMAD2-induced myocardial fibrosis (150). The activation of signal transducers and activator of transcription 3 (STAT3) has been implicated in promoting the progression of many cancers as well as exacerbating the loss of skeletal muscle tissue in cancer cachexia (151, 152). MG53 overexpression promotes the phosphorylation of STAT3, which thereby induces cardiac fibrosis, and its effect on cardiac lesions in cancer cachexia remains to be investigated (153).

Current research strongly suggests that MG53 is an important cancer therapy target, despite its potentially negative effects. Future researches should be focused on elucidating the mechanism of MG53’s role in cancer, glucose metabolism, and myogenesis, and on this basis, attempts should be made to retain the cancer therapeutic ability of MG53 while removing its side effects. MG53 mutants that retain membrane repair function without impairing glucose metabolism have been developed by eliminating the E3 ubiquitin ligase property of MG53 (128). In cancer therapy, however, the E3 ubiquitin ligase function of MG53 seems to play a crucial role. For the ubiquitination-dependent degradation of different proteins, MG53 may need to be activated at different sites, which could be the key to solving this problem (123, 128).

In summary, discussing the possible negative effects of MG53 on cancer treatment, especially in the context of varying degrees of insulin resistance and across gender, is urgently needed. If adverse effects are evident, developing safe mutants of MG53 may be a win-win approach.

## 5 Conclusions

The TRIM protein family has always been an important therapeutic target for cancer treatment, and in recent years, the role of MG53 in cancer has gradually been recognized. We found that almost all the evidence indicates that MG53 has a strong inhibitory effect on the progression of cancer and may serve as a biomarker for cancer. However, due to the lack of clinical research, the effect of MG53 on human cancer is actually undetermined. Furthermore, current research appears to overlook the contribution of the membrane repair function and anti-inflammatory properties of MG53 to cancer and does not discuss the potential adverse effects of MG53 on cancer treatment. Therefore, the safety of rhMG53 also needs further discussion. From the perspective of the biological functions of MG53, MG53 may still be a double-edged sword in cancer treatment and further research is needed to comprehensively investigate its role in cancer.

## Author contributions

YD: Writing – original draft, Writing – review & editing. TL: Writing – review & editing. MY: Supervision, Writing – review & editing.
